# Mechanical Circulatory Support Strategies in Takotsubo Syndrome with Cardiogenic Shock: A Systematic Review

**DOI:** 10.3390/jcm13020473

**Published:** 2024-01-15

**Authors:** Johanna K. R. von Mackensen, Vanessa I. T. Zwaans, Ahmed El Shazly, Karel M. Van Praet, Roland Heck, Christoph T. Starck, Felix Schoenrath, Evgenij V. Potapov, Joerg Kempfert, Stephan Jacobs, Volkmar Falk, Leonhard Wert

**Affiliations:** 1 Department of Cardiothoracic and Vascular Surgery, Deutsches Herzzentrum der Charité—Medical Heart Center of Charité and German Heart Institute, 13353 Berlin, Germany; zwaans@stud.uni-heidelberg.de (V.I.T.Z.); ahmed.el-shazly@dhzc-charite.de (A.E.S.); roland.heck@dhzc-charite.de (R.H.); christoph.starck@dhzc-charite.de (C.T.S.); felix.schoenrath@dhzc-charite.de (F.S.); evgenij.potapov@dhzc-charite.de (E.V.P.); joerg.kempfert@dhzc-charite.de (J.K.); stephan.jacobs@dhzc-charite.de (S.J.); volkmar.falk@dhzc-charite.de (V.F.); leonhard.wert@dhzc-charite.de (L.W.); 2 Department of Cardiothoracic Surgery, ASZ Hospital Aalst, 9300 Aalst, Belgium; 3 Cardiac Surgery Department, Hartcentrum OLV Aalst, 9300 Aalst, Belgium; 4 DZHK (German Center for Cardiovascular Research), Partner Site, 10785 Berlin, Germany; 5 Department of Cardiothoracic Surgery, Charité, Universitätsmedizin Berlin, Corporate Member of Freie Universität Berlin, Humboldt-Universität zu Berlin, and Berlin Institute of Health, 10117 Berlin, Germany; 6 Department of Health Sciences and Technology, ETH Zürich, 8093 Zurich, Switzerland

**Keywords:** Takotsubo, stress cardiomyopathy, cardiogenic shock, mechanical circulatory support, ECLS, IABP, Impella

## Abstract

Background: Takotsubo syndrome is, by definition, a reversible form of acute heart failure. If cardiac output is severely reduced, Takotsubo syndrome can cause cardiogenic shock, and mechanical circulatory support can serve as a bridge to recovery. To date, there are no recommendations on when to use mechanical circulatory support and on which device is particularly effective in this context. Our aim was to determine the best treatment strategy. Methods: A systematic literature research and analysis of individual patient data was performed in MEDLINE/PubMed according to PRISMA guidelines. Our research considered original works published until 31 July 2023. Results: A total of 93 publications that met the inclusion criteria were identified, providing individual data from 124 patients. Of these, 62 (50%) were treated with veno-arterial extracorporeal life support (va-ECLS), and 44 (35.5%) received a microaxial left ventricular assist device (Impella). Eighteen patients received an Impella CP and twenty-one an Impella 2.5. An intra-aortic balloon pump (IABP) without other devices was used in only 13 patients (10.5%), while other devices (BiVAD or Tandem Heart) were used in 5 patients (4%). The median initial left ventricular ejection fraction was 20%, with no difference between the four device groups except for the IABP group, which was less affected by cardiac output failure (*p* = 0.015). The overall survival was 86.3%. Compared to the other groups, the time to cardiac recovery was shorter with Impella (*p* < 0.001). Conclusions: Though the Impella treatment is new, our analysis may show a significant benefit of Impella compared to other MCS strategies for cardiogenic shock in Takotsubo syndrome.

## 1. Introduction

Takotsubo syndrome (TTS) is an acute and reversible manifestation of heart failure, commonly found in patients presenting with symptoms leading to acute coronary syndrome (ACS), especially women [1,2]. The 2019 coronavirus pandemic, as a global event with major psychosocial impacts, resulted in a dramatic increase in the number of TTS cases and further demonstrated the importance of TTS as a differential diagnosis of ACS. During the COVID-19 pandemic, the incidence of patients presenting with ACS with subsequently confirmed TTS increased to 7.8%, from 3% previously [3,4]. However, TTS remains underdiagnosed, largely due to the interplay of acute coronary syndrome.

The incidence of cardiogenic shock (CS) in patients with TTS is estimated as ranging between 6% and 20% [5,6]. Patients with TTS in CS have a high in-hospital mortality of 15% [6]. Both the Mayo Clinic Criteria [7] and the InterTAK Diagnostic Criteria [8] are used as instruments for diagnosing TTS. They include echocardiographic evidence of hypo-, a- or dyskinesia extending beyond a single coronary vascular territory, as well as exclusion by coronary angiography of coronary atherosclerosis as a causal agent of regional wall motion abnormalities. Physical or emotional triggers should additionally be detectable. The main difference between the two criteria is that, for the Mayo Clinic Criteria, the absence of pheochromocytoma must be confirmed, whereas the InterTAK Diagnostic Criteria postulate that certain neurological disorders (e.g., subarachnoid haemorrhage, stroke/transient ischaemic attack or seizures) as well as pheochromocytoma may serve as triggers for TTS [7,8]. Even though research has paid increasing attention to TTS in recent years, many aspects remain unclear. Besides its aetiology, which is not completely understood, the management of patients with TTS, especially when a severe course of stress cardiomyopathy leads to cardiogenic shock, is unclear and there is no evidence-based therapy concept available. As mechanical circulatory support (MCS) devices are increasingly used in patients with CS and are recommended for SCAI shock stages C, D and E [9], they are also used in patients with TTS with good results [10,11,12].

A so-called broken heart can be repaired mechanically. However, there are no recommendations for when MCS should be used and which device should be preferred. The literature increasingly recommends considering MCS as a bridge-to-recovery strategy in TTS-CS [13,14], which by definition is reversible, in order to minimise the use of inotropes, as these may further worsen the clinical picture, which is potentially triggered by catecholamines [15].

Data on the use of MCS in TTS-CS are rare and only retrospective. The use of IABP was evaluated in a European registry of 2248 TTS patients, of whom 43 were patients with CS treated with IABP. The data show no significant difference in 30-day mortality, length of hospitalisation, need for invasive ventilation and 42-month follow-up [16]. A review and meta-analysis of MCS for TTS-CS by Mariani et al., including data until 2019, also confirms that the use of IABP is decreasing, while the use of va-ECLS and Impella in TTS patients with CS is increasing [17]. Since the microaxial LVAD Impella is a relatively new device, its frequent use is reflected only in the most recent literature; therefore, the review from Mariani et al. from 2020 included only 10 patients supported by Impella. The Impella is a non-durable microaxial left ventricular assist device (LVAD). Different surgical Impella procedures exist: the implantation of an Impella CP in combination with veno-arterial ECLS via the femoral artery is called ECMELLA 1.0 [18], while the combination of a single axillary artery approach for an Impella CP with veno-arterial ECLS is referred to as ECMELLA 2.0 [19]. The various procedures are associated with different complications (e.g., wound infection rates) and possibilities (e.g., early patient mobilisation) [20]. In recent years, it has been increasingly suggested that microaxial LVADs may be of particular value in the treatment of CS caused by TTS, as they enable venting of the left ventricle [21,22,23]. There is thus a growing interest to evaluate patients with TTS-CS who have been treated with Impella [24,25,26,27].

A retrospective multicentre analysis conducted in 2022 evaluated 16 patients with TTS-CS treated with Impella; the data indicated favourable outcomes, with good survival and full recovery of LV function [24].

In general, the literature contains reports on the use of intra-aortic balloon pumps (IABP), TandemHeart, extracorporeal membrane oxygenation (ECLS) and microaxial pumps (i.e., Impella) for the treatment of TTS-CS. Since interest in this clinical cardiac entity is increasing, there is also a rapidly growing number of published case reports and series relating to the use of MCS devices in TTS-CS. To recognise the trends and outcomes of this currently rapidly growing population of patients with TTS-CS treated with MCS and to compare the different support systems that can be applied, we performed a systematic review and analysis of the existing literature.

## 2. Materials and Methods

This review was conducted and reported in accordance with the Preferred Reporting Items for Systematic Reviews and Meta-Analyses (PRISMA) guidelines [28]. Prior to the literature review, the concept, inclusion criteria, research question and hypothesis were defined. The aim was to compare the different devices used so far as MSC strategies in the context of TTS-CS in the existing literature. The underlying hypothesis was that left ventricular venting and physiological antegrade support provided by the microaxial Impella device allows the overloaded ventricle to recover faster and that MCS is therefore needed for a shorter period of time.

### 2.1. Search Strategies

**Figure 1:**
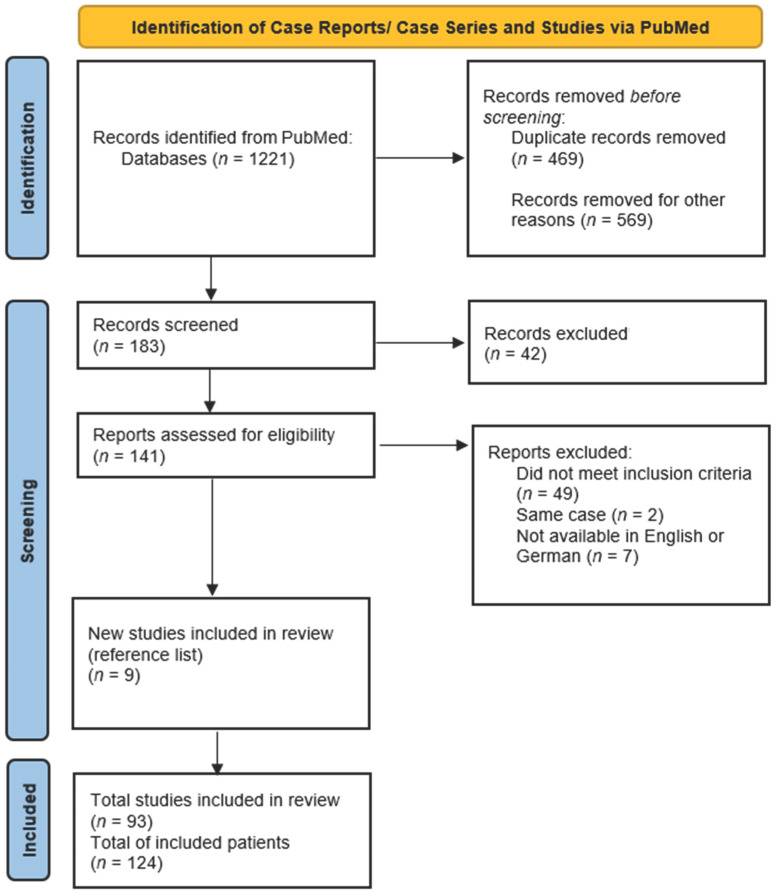
PRISMA (Preferred Reporting Items for Systematic Review and Meta-Analysis) flowchart of the systematic literature review [29].

A systematic search in PubMed of articles published until 31 July 2023 was carried out using the following mesh terms: (‘takotsubo’ or ‘tako-tsubo’ or ‘stress cardiomyopathy’ or ‘apical ballooning’) AND (‘ECLS’ or ‘ECMO’ or ‘Impella’ or ‘IABP’ or ‘mechanical support’ or ‘mechanical circulatory support’ or ‘MCS’ or ‘assist device’). The titles of the initial results were screened and all publications matching the inclusion criteria of confirmed TTS and use of MCS were imported into the reference management software EndNote 20^®^ (Clarivate Analytics, London, UK). Duplicates were removed and the remaining publications were assessed for inclusion and screened for additional matching references. Full texts of all relevant articles were obtained and evaluated by the first author (Figure 1).

### 2.2. Inclusion Criteria

Published case reports and case series as well as retrospective, observational or randomised studies among patients diagnosed with TTS were considered. Inclusion required documentation of the following criteria:

1. TTS diagnosis consistent with the InterTAK Diagnostic Criteria and designated as such by the authors;
2. Use of temporary MCS: IABP, ECLS, Impella, TandemHeart;
3. Individually reported patient data in terms of pre-intervention status, survival and time on support.

Studies not written in English or German, poorly described cases and review articles were excluded. Case series or studies not providing primary data or where the analysis was pooled without a description of individual patient data were excluded.

### 2.3. Data Extraction

**Figure 2:**
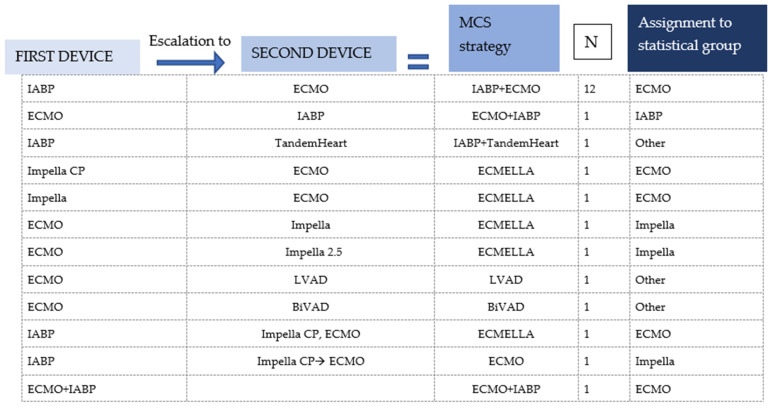
Assignment to statistical group/single-device group of patients who were treated with more than one device and with a combination of two devices or where the first device was removed and replaced by another.

Data were extracted by the first author and independently checked for accuracy by the last author. Data extracted included authors, title, year of publication, patient demographics (age, sex), MCS strategy, vital signs before circulatory support (systolic/diastolic blood pressure or MAP, heart rate), respiratory failure, use of inotropes before MCS, performed angiography (LVEDP), cardiac arrest before MCS, new ECG abnormalities (ST elevation, ST depression and T wave inversion), new arrhythmias, LVEF (before MCS, on MCS and in follow-up), NTproBNP/BNP (baseline and follow-up), baseline lactate, presence of left ventricular outflow tract obstruction (LVOTO) (gradient) or mitral regurgitation (MR) (systolic anterior motion), trigger, TTS type (inverted or normal), RV involvement, time to weaning of inotropes under MCS, time on MCS and follow-up (last documented date after implantation, LV recovery and survival). Patients treated with more than one device were assigned to a device group for statistical analysis (see Figure 2). The assignment was based on the device to which the authors attributed therapy success. Thus, if MCS therapy was escalated to another device under which therapy success was achieved, the patient was assigned to the device group to which it was escalated.

### 2.4. Statistics

Descriptive statistical analysis was performed by using the data analysis tools IBM^®^ SPSS 29 (IBM, Armonk, New York, NY, USA) and R, Version 4.02.(R Project for Statistical Computing, the R Foundation for Statistical Computing, Vienna, Austria).

The following data were collected: median with interquartile range (IQR) or mean ± SD for the total group and for the separate device subgroups. Categorical variables are presented as *N* (%) and were compared using Fisher’s exact test. Metric data were analysed for >2 independent variables using the Friedman test or the Kruskal–Wallis test depending on the distribution (normal or non-normal). A two-sided *p*-value lower than 0.05 was considered statistically significant. To enable assignment of significant results, a pairwise comparison was performed if more than two variables (e.g., four device groups) were used.

## 3. Results

### 3.1. Literature Research

The search in PubMed yielded a total of 1221 publications. A screening of the titles identified 1038 irrelevant or double publications. A total of 183 articles were screened on the basis of the abstract; of these, 141 were classified as relevant and full-text reading was carried out. A screening of the reference list yielded a further nine publications. Forty-nine articles did not meet the inclusion criteria or provide relevant information and were therefore excluded. Two articles depicted the same case, so one was excluded [30,31]. Seven articles could not be included because the full text was not available in English or German [32,33,34,35,36,37,38].

**Figure 3:**
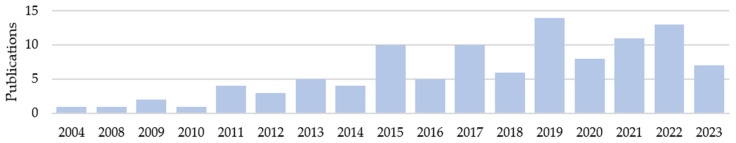
Number of included publications by year.

After full-text evaluation, 93 articles providing individual data on 124 cases were included (Appendix A). While the publications cover a period of almost 20 years, the majority were from the last five years (Figure 3).

The quality of case-related data in the publications varied widely. In particular, some articles were dedicated to specific aspects, e.g., echocardiographic control or therapy of the underlying condition, and therefore provided little general data. Overall, an almost complete data set could be collected for demographics, baseline LVEF and the duration of mechanical support. Especially information regarding follow-up and clinical status prior to the establishment of MCS, e.g., blood pressure, heart rate or catecholamines before MCS, varied greatly in terms of availability and quality. Unfortunately, important haemodynamic parameters such as right heart catheter findings or parameters of extended cardiac monitoring were not provided in the underlying literature.

### 3.2. Demographics and Device Groups

A data set of 124 patients was obtained, 92 of whom were female (74.2%). Five paediatric cases were included (age < 18 years). The median age was 52.2 years (Table 1).

**Figure 4:**
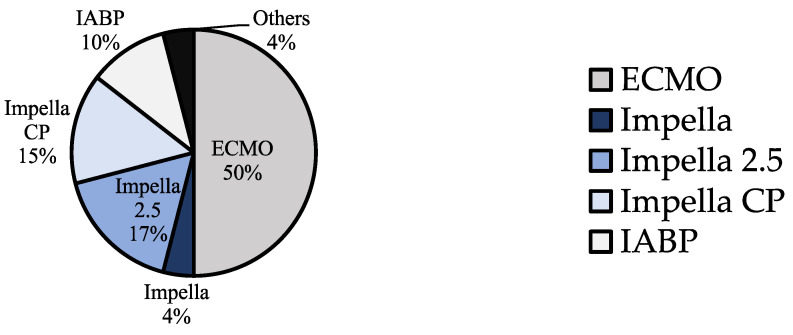
Percentage of the different device groups (ECLS, Impella, Impella 2.5, Impella CP, IABP and Other) in the total collective of 124 patients with TTS and CS treated with MCS.

After assigning the patients with two or more devices to a device group (Figure 2), the patient collective was divided into four device groups: ECLS, Impella, IABP and Other (Figure 4). The ‘Other’ group included two cases where a TandemHeart was combined with IABP and Impella, as well as one case where central ECLS was established and two cases treated with a BiVAD [39,40,41,42,43]. The Impella group was further subdivided according to the generation used: the Impella 2.5, which can increase cardiac output (CO) by about 2.5–3 L/min, was the most frequently used model, accounting for 47.7% of Impella cases. The Impella CP, which can relieve the left ventricle and support the circulation with a higher capacity of up to 4 L/min, was used in 40.9% of the cases. In 4% of the Impella cases, no exact model was named.

### 3.3. Pre-MCS Status of the Patients

In most cases, TTS was caused by physical triggers, but there were also cases in which a combination of physical and emotional triggers was present. Iatrogenic catecholamine administration was thought to be the cause of TTS-CS in six patients [40,44,45,46,47]. Nineteen patients developed TTS-CS in the context of pheochromocytoma [48,49,50,51,52,53,54,55,56,57,58,59,60], all of whom received ECLS as MCS, one in combination with IABP and one in combination with Impella. Due to pheochromocytoma, these patients exhibited a markedly higher blood pressure before MCS, with a mean systolic blood pressure of 140.9 mmHg. This is also reflected in the difference in the median systolic blood pressures between the different device groups, with the ECLS groups showing a median systolic blood pressure of 95 mmHg compared with 82 mmHg or lower in the other groups. Another difference in pre-implantation status between the groups was the frequency of cardiac catheterisation to rule out coronary obstruction. This is presumably attributable to the fact that both IABP as well as Impella 2.5 and CP are placed under angiographic support, which means that these patients inevitably visit the catheter laboratory; catheterisation is thus also mentioned in almost every case report. Another important difference is the presence of pulmonary oedema or respiratory failure. Patients who experienced a pulmonary complication or respiratory failure during CS more frequently received ECLS. There were no significant differences between the device groups with regard to cardiac arrest, ST elevations in the electrocardiogram (ECG), need for inotropes before implantation or elevated troponin levels.

Data between the groups regarding the clinical status of the patients before MCS therapy vary only for some parameters and are generally comparable (Table 2). However, for several parameters, data are only available from a fraction of the patients, making it difficult to draw conclusions as to the comparability of the groups. Regarding pre-implantation status, baseline LVEF is considered a key parameter for assessing the severity of cardiac compromise and the comparability of the severity of pump failure between the groups.

It should be emphasised that there was no significant difference in baseline LVEF across the various MCS groups. With a *p* value of 0.015, a significant result was found when comparing the median baseline LVEF values between the groups; however, a pairwise comparison showed that this significance existed only between IABP and the Impella as well as IABP and ECLS. The median values of baseline LVEF between the Impella and ECLS, however, did not differ significantly (*p*: 0.665).

### 3.4. Outcome and Follow-Up

**Figure 5:**
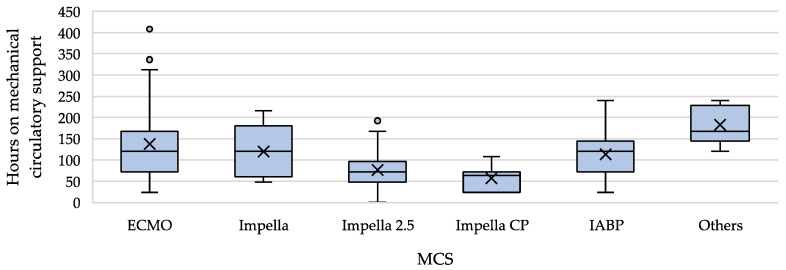
Comparison of mechanical circulatory support duration with median, IQR, outliers and mean (=x). For the number of cases evaluated per group, see Table 3.

In conclusion, the outcomes were good, with an overall survival rate of 86.3%. It should also be emphasised that in those cases where the patients died, they mostly suffered from very severe underlying diseases (two perioperative cases after liver transplant, one perioperative case after double-lung transplant, three cases with pheochromocytoma, two cases after polytrauma, one case with severe hypothermia and hypoglycaemia) [61,62,63,64,65,66]. In three cases, the authors did not attribute the patient’s death directly to the underlining condition. One suffered from advanced motor neuron disease and developed pneumonia and sepsis during MCS. One developed TTS after mitral valve reconstruction surgery. One case ‘merely’ experienced an emotional trigger but suffered from severe TTS; this patient died from the complications of MCS [67,68,69]. Significant differences between the groups, not caused by insufficient or inconsistent data (length of follow-up), were found only with regard to median support duration. Here, the Impella device clearly showed significantly shorter support times, which were also confirmed in pairwise comparison with every other device. Especially interesting is that the analysis of the Impella subgroups regarding the duration of MCS (Figure 5) showed that the duration of support decreased with the performance of the Impella generation. In other words: the greater the degree of LV venting, the faster the heart recovers and mechanical support can be terminated.

Unfortunately, it was hardly ever reported whether complications occurred during MCS therapy (Table 3). Furthermore, the recorded complications are not based on standardised definitions, but rather on what the authors of the primary literature report. In total, MCS complications were reported in 36 patients (29%). The occurrence of complications did not differ significantly between the groups (*p* = 0.927). Since several specific complications were mentioned in some cases and these were recorded, more individual complications are listed in some device groups in Table 4 than for the total number of patients suffering from complications in the device group. In a few publications, the persistence of cardiogenic shock under MCS was considered a complication, but because all patients in whom therapy was escalated to another device theoretically suffered from persistent CS under MCS, this category is not presented. Since the individual complication items are not based on common definitions, but merely on the fact that the authors of the case reports referred to them as such, and since complications under MCS were not mentioned in many articles, we deliberately refrained from performing a statistical evaluation of these parameters.

### 3.5. Comparison of the Device Groups

**Figure 6:**
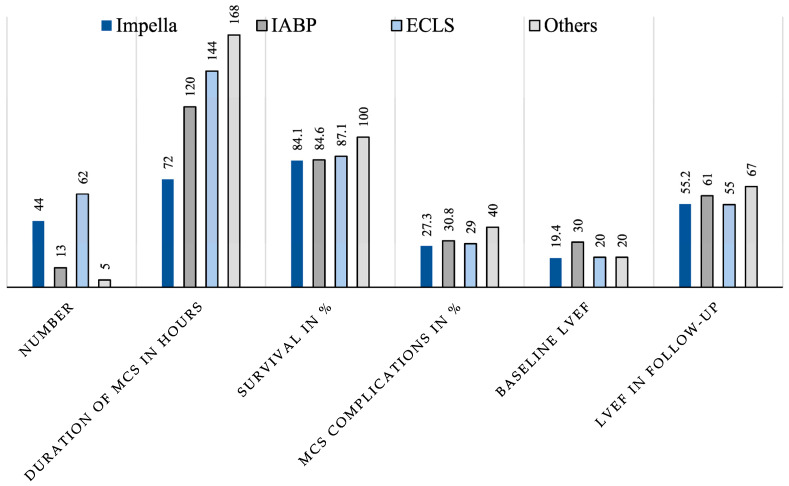
Graphical representation of important parameters for comparing the four different device groups (Impella, IABP, ECMO, Other).

The collected data were analysed statistically with regard to the hypothesis that due to the pathophysiology of TTS-CS and the function of the different devices, the Impella is particularly suitable for circulatory support in TTS-CS. The data revealed that with comparable disease severity (especially baseline LVEF), patients in the Impella group required MCS for a significantly shorter period of time. However, there were no clear differences in the complication rates and overall survival (Figure 6).

## 4. Discussion

The patient population with Takotsubo syndrome is a very heterogeneous group, both in terms of concomitant diseases and the triggers of TTS. Some patients develop TTS within the scope of a severe non-cardiac condition and therefore have a critical status simply because of their underlying disease. Other patients, however, develop TTS within the scope of an emotional stressor and are physically healthy until they develop TTS. In addition, the diagnostic criteria that can be applied differ with respect to the inclusion of patients who develop TTS in the context of a neurological disease or pheochromocytoma. These patients are included when the InterTAK criteria are applied, thus generating a much more heterogeneous patient population, even if cardiac shock develops in the context of TTS, since the usual parameters such as low systolic pressure cannot be used to assess the severity of the clinical status in patients with pheochromocytoma. In addition to the very heterogeneous patient population, this evaluation is grounded on a very heterogeneous and retrospective data base. Despite the retrospective and heterogeneous character of the data, there are indications that the data collected are representative, as they match existing data in many aspects. For example, a mortality of about 15% is described for patients with TTS-CS [3,7]. In the present data, the mortality was 13.7%. Other parameters such as the frequency of ECG changes or an increase in cardiac markers are also consistent with data collected in larger registries [7,13,70,71].

Concerning the question of which device might be appropriate for treating CS in TTS, the following considerations regarding the pathophysiology of TTS and the mechanisms of the devices can be made and are discussed in the literature underlying this review.

The intra-aortic balloon pump (IABP) works with counterpulsation. This therapy is only able to increase the cardiac index to a limited extent and can worsen or even cause LVOTO [72]. Another argument against IABP is that the rapid heartbeat that is frequently present in TTS-CS may be too fast for IABP to follow [73]. Furthermore, some studies suggest that IABP shows no advantage over optimal drug therapy without mechanical circulatory support [11,17].

Veno-arterial extracorporeal membrane oxygenation (v-a ECLS) is often used with the argument that the patient is suffering from respiratory failure on top of uncompensated acute heart failure despite inotropic and ventilatory support. Other reasons for the use of ECLS are that it also can be applied in children or patients who are too short for Impella [74,75].

One reason against using v-a ECLS is that, due to providing retrograde circulatory support, it increases LV afterload, which can further increase the already elevated left ventricular end-diastolic pressure (LVEDP).

The microaxial Impella pump provides physiological cardiac support which improves coronary and end-organ perfusion while simultaneously unloading the left ventricle. The main advantages of the Impella in TTS-CS are that cardiac output can be increased simultaneously with venting of the overloaded LV, thereby improving pulmonary congestion by reducing LV preload and increasing cardiac output regardless of the heart rate. Also, in several reports, the availability of the Impella was given as the reason for choosing this device [26,28,52,53,76,77].

The positive effect of LV unloading becomes apparent when comparing patients with CS treated with ECLS alone or with ECMELLA. LV unloading has been associated with a lower 30-day mortality [22].

In order to interpret the outcome of the different device groups in this analysis, the gathered data must first be critically analysed with regard to their quality and comparability. Some parameters were only reported for a fraction of the patients in the primary literature. Other parameters, such as the categorisation of the trigger as emotional or physical, are not based on hard criteria, but on what the authors of the case reports write. The achievement of complete recovery is also a descriptive parameter, which was collected from the texts of the underlying literature and is not based on defined clinical parameters. However, some of the parameters were well reported: baseline LVEF was available for 76.6% of the patients, and the duration of mechanical circulatory support was available for 91.1% of patients. Other parameters that would have led to a more precise comparability of the groups were, unfortunately, not available at all.

It can be assumed that the patients who received the Impella for the treatment of CS in the context of TTS were equally affected by pump failure, represented by reduced LVEF, which was comparable between the groups; only the IABP group was significantly different with a better LVEF. Therefore, for the other groups, we can assume that the level of cardiac compromise was comparably severe. Additional other parameters such as NTproBNP, elevated troponin and cardiac arrest also indicate a comparable disease severity between the groups. The Impella group showed a significantly shorter duration of mechanical circulatory support. This finding, along with a comparable disease severity within the Impella group, may suggest that left ventricular venting is relevant for a rapid recovery from TTS.

Several publications about TTS-CS demonstrate high survival and recovery rates, also since TTS is, by definition, a form of temporary heart failure [4,7]. Therefore, when dealing with cardiogenic shock in the context of TTS, MCS can be used as part of a bridge-to-recovery concept. As the literature also clearly shows that the complications of MCS increase with time on support, MCS therapy should be administered for as short a time as possible to avoid complications [19]. For this reason, microaxial LVADs may be the best option for treating CS in TTS. It could therefore be assumed that in the present data, with significantly shorter support durations in the Impella group, significantly lower complication rates would also be expected. However, this was not the case. One reason for this could be that the Impella group also included data from the multicentre study of Impella use in TTS-CS by Napp et al., which also provides detailed data on the type and frequency of complications, unlike the majority of the case reports, which rarely focus on or even mention MCS complications. This demonstrates the urgent need for prospective multicentre studies investigating the use of MCS devices in TTS-CS with a critical eye on complications associated with MCS.

The available data are also insufficient to make a statement on the benefit of one device with regard to the recovery presented in the follow-up. A complicating factor here is that the underlying case reports often do not provide any information about the follow-up period or the clinical parameters that demonstrate recovery. In some cases, it was only described that complete recovery was achieved and that the patient was discharged home.

In conclusion, the data suggest that unloading the ballooned ventricle accelerates myocardial recovery, and that preference should be given to devices that increase neither the gradient across the LVOT nor the LVEDP. To provide evidence for the treatment of CS induced by TTS, prospective and multicentre studies investigating different MCS devices as well as mechanical versus conservative shock therapies in this specific setting are urgently needed.

## 5. Limitations

Three main points can be highlighted which limit the results of this systematic review. First, the heterogeneous data base: our systematic review represents the largest population of patients with mechanical circulatory support for CS caused by TTS to date. It is based predominantly on case reports, which are often very detailed but usually focus on a specific question and therefore do not always represent all parameters in precisely the same manner. There are also no standardized criteria for some parameters, such as a patient’s full recovery. Parameters such as the frequency of catheterization prior to MCS implantation clearly show the weakness of an analysis based on such heterogenic baseline data. In the case of an exclusion diagnosis such as TTS, a very high proportion of catheterizations would be expected to rule out ACS. However, catheterization prior to MCS is reported in only 66.9% of cases, which clearly appears too low and exemplifies the limitations of the present work.

Second, this analysis is retrospective and covers a period of 20 years. During this period, MCS technology improved and also became more widely available. This may also have changed the potential patient population in whom MCS could be applied in the setting of TTS-CS. Since the SCAI classification was established in 2019, the SCAI stage was not documented in the individual case reports, not least because the literature included in the present analysis covers a period of 20 years. Furthermore, the retrospective nature of this study poses a challenge for the inclusion of studies. The diagnosis of TTS and CS declared by the authors had to be verified on the basis of the data available in the publications (fulfilment of the InterTak diagnostic criteria and haemodynamic as well as clinical criteria of CS). If no additional information was available that would allow the assessment of the InterTak score to determine whether cardiogenic shock caused by takotsubo syndrome was a plausible diagnosis, studies were not included.

Third, when looking at the differences between the various devices, the following must be considered: there is a high cross-over rate between devices in the data set, which reduces the separation between the groups and biases this review towards finding no difference. Furthermore, the conclusion that MCS with the Impella is significantly better than other MCS strategies was based mainly on the parameters of baseline LVEF and duration of MCS. To confirm that the correlations identified here are causal and to clearly show that left ventricular ventilation leads to faster myocardial recovery, prospective multicentre studies are needed that also consider more precise parameters.

## 6. Conclusions

This systematic review and pooled analysis of individual data involved 124 patients with TTS-CS treated with MCS. Cardiac recovery was shorter with the Impella, with no differences in terms of survival and full recovery of left ventricular function. The superiority of microaxial LVADs over the other devices can merely be assumed on the basis of the shorter duration of mechanical support. Prospective randomized studies are needed to provide recommendations on the use of MCS in TTS-CS.

## Figures and Tables

**Table 1:** Demographic characteristics (age and sex). Proportions of the Impella 2.5 and Impella CP subgroups in the Impella group. Percentage of patients in whom inverted TTS was described and percentage of patients in whom the right ventricle was also involved.

	ECMO	Impella	IABP	Others	All	*p*
*N*	62	44	13	5	124	
Impella CP		18 (40.9%)				
Impella 2.5		21 (47.7%)				
Age, median (IQR)	45 (31–56)	68 (55–76)	67.5 (48–69.75)	46 (26–53)	52.2	
Sex category						0.275
Female	42 (67.7%)	34 (77.3%)	12 (92.3%)	4 (80%)	92 (74.2%)	
Male	20 (32.3%)	10 (22.7%)	1 (7.7%)	1 (20%)	32 (25.8%)	
Inverted TTS	17 (27.4)%	4 (9.1%)	2 (15.4%)	1 (20%)	24 (19.4%)	0.099
RV involved	6 (9.7%)	1 (2.3%)	1 (7.7%)	1 (20%)	9 (7.3%)	0.205

**Table 2:** Clinical status before MCS implantation. Metric data are reported as mean with IQR (interquartile range) and number of available data (*n*). Nominal data are given in number of patients and % of the device group.

	ECLS	Impella	IABP	Others	All	*p*
Initial LVEF, median (IQR)	20% (15–24) (*n* = 43)	19.4% (19.4–21.35) (*n* = 38)	30% (22.5–33) (*n* = 9)	20% (9.5–28.5) (*n* = 5)	20% (*n* = 95)	0.015
Inotropes before MCS	47 (75.8%)	35 (79.5%)	11 (84.6%)	5 (100%)	98 (79%)	0.876
Respiratory failure	43 (69.4%)	13 (29.5%)	3 (23.1%)	2 (40%)	61 (49.2%)	<0.001
Arrhythmia	19 (30.6%)	14 (31.8%)	6 (46.2%)	3 (60%)	42 (33.9%)	0.002
ST elevation	24 (38.7%)	22 (50%)	5 (38.5%)	2 (40%)	53 (42.7%)	0.88
ST depression	10 (16.1%)	5 (11.4%)	3 (23.1%)	0	18 (14.5)	0.002
T-wave inversion	16 (25.8%)	6 (13.6%)	4 (30.8%)	0	26 (21%)	0.002
Troponin elevation	44 (71%)	24 (54.5%)	11 (84.6%)	2 (40%)	81 (65.3%)	0.067
Cardiac arrest	23 (37.1%)	9 (20.5%)	3 (32.1%)	2 (40%)	37 (29.8%)	0.081
Angiography before MCS	31 (50%)	41 (93.2%)	10 (76.9%)	1 (20%)	83 (66.9%)	<0.001
NTproBNP, mean	8293.5 (*n* = 13)	13,697.4 (*n* = 5)	1832.0 (*n* = 3)	22,842 (*n* = 1)	9301.85 (*n* = 22)	0.089
BNP, mean	6342.3 (*n* = 8)	946 (*n* = 3)	4900.0 (*n* = 1)	*n* = 0	4873 (*n* = 12)	
BP syst in mmHg, median (IQR) /MAP in mmHg, mean	95 (79–141) (*n* = 35) /59.5 (*n* = 2)	80 (73–100) (*n* = 31) /50 (*n* = 3)	82 (80–115) (*n* = 6) /51.5 (*n* = 4)	74 (*n* = 2) /(*n* = 0)		0.134/0.399
HR median (IQR)	129.5 (100.25–148.75) (*n* = 32)	126 (106.25–136.75) (*n* = 28)	127 (111–147) (*n* = 4)	121 (*n* = 2)		0.933
LVEDP, median (IQR)	25 (*n* = 3)	26 (23–30) (*n* = 11)	12 (*n* = 2)	(*n* = 0)		0.357
Initial lactate, median (IQR)	69.8 (52.6–116.2) (*n* = 18)	44.1 (27.5–57.6) (*n* = 22)	104 (*n* = 2)	(*n* = 0)		0.038
Pheochromocytoma	17	1 (ECMELLA *n* = 2)	(ECLS + IABP: *n* = 1)		19	
Mitral regurgitation	7 (11.3%)	5 (11.4%)	2 (15.4%)	1 (20%)	15 (12.1%)	0.662
Systolic anterior motion	3 (4.8%)	5 (11.4%)	2 (15.4%)	0	10 (8.1%)	
LVOTO	3 (4.8%)	7 (15.9%)	2 (15.4%)	0	12 (9.7%)	0.174
Emotional trigger	16 (25.8%)	17 (38.6%)	15.9 (53.8%)	4 (80%)	44 (35.5%)	0.002
Physical trigger	58 (93.5%)	22 (50%)	11 (84.6%)	4 (80%)	95 (76.6%)	<0.001
Exogenous catecholamine trigger	4 (6.5%)	1 (2.3%)	1 (7.7%)	0	6 (4.8%)	0.583

**Table 3:** Outcome and follow-up data presented as metric data reported as means with IQRs (interquartile ranges) and number of available data (*n*). Nominal data given in number of patients and % of device group. Two side *p*-value of the statistical comparison of the different device groups.

	ECLS	Impella	IABP	Others	All	*p*
Survival in %	54 (87.1%)	37 (84.1%)	11 (84.6%)	5 (100%)	107 (86.3%)	0.855
Hours on MCS, median (IQR)	144 (72–192) (*n* = 55)	72 (48–96) (*n* = 42)	120 (72–144) (*n* = 11)	168 (144–228)	96 (*n* = 113)	<0.001
MCS complications	18 (29%)	12 (27.3%9)	3.8 (30.8%)	2 (40%)	36 (24.2%)	0.927
Follow-up	45 (72.6%)	21 (47.7%)	9 (69.2%)	4 (80%)	79 (63.7%)	0.049
Max. follow-up in weeks, median (IQR)	8 (4–24) (*n* = 45)	4 (2–8) (*n* = 19)	2 (1.75–9) (*n* = 9)	28 (18–44) (*n* = 4)	6 (*n* = 77)	0.01
LVEF in follow-up, median (IQR)	55% (55–65) (*n* = 22)	55.2% (*n* = 22)	61% (*n* = 7)	67% (57–72) (*n* = 5)	58.5% (*n* = 56)	0.262
Full recovery	47 (75.8%)	32 (72.7%)	8 (61.5%)	5 (100%)	92 (74.2%)	0.458

**Table 4:** Frequency of reported complications (multiple mentions per patient possible).

	ECLS	Impella	IABP	Others
Complications under MCS	18 (29%)	12 (27.3%)	4 (30.8%)	2 (40%)
Stroke	1 (1.6%)	0	0	0
Prolonged weaning of MCS	2 (3.2%)	0	0	0
Pericardial tamponade	2 (3.2%)	0	0	0
MR development under MCS	0	1 (2.3%)	0	0
Ventricle rupture	0	1 (2.3%)	0	0
Development of atrial fibrillation under MCS	1 (1.6%)	1 (2.3%)	0	0
Haemolysis	0	3 (6.8%)	0	0
Significant HB drop/bleeding	4 (6.5%)	5 (11.4%)	1 (7.7%)	0
Significant thrombocytopenia	3 (4.8%)	3 (6.8%)	0	0
AKI	3 (4.8%)	3 (6.8%)	0	0
Vascular damage in the lower limb	5 (8.1%)	1 (2.3%)	1 (7.7%)	0
Vascular damage in the upper limb	1 (1.6%)	0	0	0
Death while on support	0	1 (2.3%)	2 (15.4%)	0

## Data Availability

All data are available, see Appendix A.

## Glossary of Abbreviations

LV	left ventricle/left ventricular
LVAD	left ventricular assist device
BiVAD	biventricular assist device
ECMO	extracorporeal membrane oxygenation
ECLS	extracorporeal life support
IABP	intra-aortic balloon pump
LVEF	left ventricular ejection fraction
NYHA	New York Heart Association
TTS	Takotsubo syndrome
CS	cardiogenic shock
ACS	acute coronary syndrome
MCS	mechanical circulatory support
MR	mitral regurgitation
SAM	systolic anterior motion
MAP	mean arterial pressure
CO	cardiac output
AKI	acute kidney injury
HB	haemoglobin

## Appendix A. Included Publications

**Author**	**Journal**	**Year**	**Title**	**Number of Patients**
A. A. Oredegbe and M. Awad	Cureus	2023	Catecholamine Mega Storm Triggered by Cocaine Use and Thyrotoxicosis Crisis	1
A. Badouin and O. Bastien	Anaesth Crit Care Pain Med	2015	ECLS indication for a case of stress myocardiopathy associated with severe asthma	1
A. Benak, M. Sramko, B. Janek et al.	Cureus	2022	Successful Treatment of Cardiogenic Shock Due to Takotsubo Cardiomyopathy With Left Ventricular Outflow Tract Obstruction and Acute Mitral Regurgitation by Impella CP	1
A. Beneduce, L. Fausta Bertoldi, F. Melillo et al.	JACC Cardiovasc Interv	2019	Mechanical Circulatory Support With Impella Percutaneous Ventricular Assist Device as a Bridge to Recovery in Takotsubo Syndrome Complicated by Cardiogenic Shock and Left Ventricular Outflow Tract Obstruction	1
A. H. Koop, R. E. Bailey and P. E. Lowman	BMJ Case Rep	2018	Acute pancreatitis-induced Takotsubo cardiomyopathy and cardiogenic shock treated with a percutaneous left ventricular assist device	1
A. K. Tiwari and N. D’Attellis	J Cardiothorac Vasc Anesth	2008	Intraoperative left ventricular apical ballooning: transient Takotsubo cardiomyopathy during orthotopic liver transplantation	1
A. Lauterio, M. Bottiroli, A. Cannata et al.	Minerva Anestesiol	2022	Successful recovery from severe inverted Takotsubo cardiomyopathy after liver transplantation: the efficacy of extracorporeal membrane oxygenation (ECMO)	1
A. Mohammedzein, A. Taha, A. Salwan et al.	JACC Case Rep	2019	Impella Use in Cardiogenic Shock Due to Takotsubo Cardiomyopathy With Left Ventricular Outflow Tract Obstruction	1
A. Omosule, M. F. Malik, L. Cisneros et al.	J Cardiothorac Vasc Anesth	2019	Takotsubo Cardiomyopathy After Double-Lung Transplantation: Role of Early Extracorporeal Membrane Oxygenation Support	1
A. Rashed, S. Won, M. Saad et al.	BMJ Case Rep	2015	Use of the Impella 2.5 left ventricular assist device in a patient with cardiogenic shock secondary to Takotsubo cardiomyopathy	1
A. Vachiat, K. McCutcheon, A. Mahomed et al.	Cardiovasc J Afr	2016	Takotsubo cardiomyopathy post liver transplantation	1
A. Yazicioglu, M. Subasi, S. Turkkan et al.	Turk Gogus Kalp Damar Cerrahisi Derg	2018	An uncommon cause for grade 3 primary graft dysfunction after lung transplantation: Takotsubo cardiomyopathy	1
B. F. Sagger Mawri, Hazem Malas, Sachin Parikh et al.	Scient Open Access	2017	Mechanical Hemodynamic Support as a Bridge to Recovery in Severe Takotsubo Cardiomyopathy with Marked Left Ventricular Outflow Tract Obstruction and Cardiogenic Shock	1
B. Flam, M. Broome, B. Frenckner et al.	J Intensive Care Med	2015	Pheochromocytoma-Induced Inverted Takotsubo-Like Cardiomyopathy Leading to Cardiogenic Shock Successfully Treated With Extracorporeal Membrane Oxygenation	1
B. Hassid, S. Azmoon, W. S. Aronow et al.	Arch Med Sci	2010	Hemodynamic support with TandemHeart in tako-tsubo cardiomyopathy—a case report	1
B. Laliberte and B. N. Reed	Am J Health Syst Pharm	2017	Use of an argatroban-based purge solution in a percutaneous ventricular assist device	1
B. M. M. Faria, J. Portugues, R. Roncon-Albuquerque et al.	Eur Heart J Case Rep	2020	Inverted Takotsubo syndrome complicated with cardiogenic shock requiring veno-arterial extracorporeal membrane oxygenation in a patient with bilateral pheochromocytoma: a case report	1
C. Dominedo, E. D’Avino, A. Martinotti et al.	Eur Heart J Case Rep	2021	A rare pheochromocytoma complicated by cardiogenic shock and posterior reversible encephalopathy syndrome: case report	1
C. J. van Zwet, A. Rist, A. Haeussler et al.	A A Case Rep	2016	Extracorporeal Membrane Oxygenation for Treatment of Acute Inverted Takotsubo-Like Cardiomyopathy From Hemorrhagic Pheochromocytoma in Late Pregnancy	1
C. Zeballos, R. J. Moraca, S. H. Bailey et al.	J Card Surg	2012	Temporary mechanical circulatory support for Takotsubo cardiomyopathy secondary to primary mediastinal B-cell lymphoma	1
D. Basic, G. Klug, B. Haubner et al.	Eur Heart J	2019	Left ventricular unloading by percutaneous mechanical circulatory support in Takotsubo syndrome with severe cardiogenic shock	1
D. Ghanim, Z. Adler, D. Qarawani et al.	J Med Case Rep	2015	Takotsubo cardiomyopathy caused by epinephrine-treated bee sting anaphylaxis: a case report	1
D. Laghlam, O. Touboul, M. Herry et al.	Front Cardiovasc Med	2022	Takotsubo cardiomyopathy after cardiac surgery: A case-series and systematic review of literature	2
D. W. Donker, E. Pragt, P. W. Weerwind et al.	Int J Cardiol	2012	Rescue extracorporeal life support as a bridge to reflection in fulminant stress-induced cardiomyopathy	1
E. Barsoum, S. Elhosseiny, B. Patel et al.	Heart Lung	2021	Successful use of the impella ventricular assist device for management of reverse Takotsubo Cardiomyopathy in the setting of acute intracranial hemorrhage	1
E. C. Busse and J. M. Wiater	JBJS Case Connect	2015	Perioperative Takotsubo Cardiomyopathy: A Rare Cardiac Complication Following Orthopaedic Surgery: A Case Report	1
E. D Foley, Ricardo Diaz and Manuel R Castresana	SAGE Open Med Case Rep	2017	Prolonged circulatory support with an Impella assist device in the management of cardiogenic shock associated with Takotsubo syndrome, severe sepsis and acute respiratory distress syndrome	1
E. f. J. Hamid T, Fraser D, Fath-Ordoubadi F	J Clin Exp Cardiolog	2013	Use of the Impella Left Ventricular Assist Device as a Bridge to Recovery in a Patient with Cardiogenic Shock Related to Takotsubo Cardiomyopathy.	1
E. T. Wu, T. H. Lin, C. H. Lin et al.	Taiwan J Obstet Gynecol	2014	Left ventricular assist device for stress-induced cardiomyopathy after postpartum hemorrhage	1
F. F. Zhou, J. S. Ding, M. Zhang et al.	Open Med (Wars)	2022	Paraganglioma-induced inverted Takotsubo-like cardiomyopathy leading to cardiogenic shock successfully treated with extracorporeal membrane oxygenation	1
F. Yazdi, M. Blackmon, A. Kattubadi et al.	Cureus	2023	Seizure-Induced Cardiomyopathy: A Case of Takotsubo Cardiomyopathy Following an Epileptic Event	1
F. Zilio, S. Muraglia and R. Bonmassari	European Heart Journal—Case Reports	2021	Cardiac arrest complicating cardiogenic shock: from pathophysiological insights to Impella-assisted cardiopulmonary resuscitation in a pheochromocytoma-induced Takotsubo cardiomyopathy-a case report	1
G. Caturegli, M. A. Crane, E. Etchill et al.	ASAIO J	2022	Stress-Induced (Takotsubo) Cardiomyopathy After Liver Transplant Rescued with Venoarterial Extracorporeal Membrane Oxygenation	1
G. Hekimian, F. Kharcha, N. Brechot et al.	Ann Intensive Care	2016	Extracorporeal membrane oxygenation for pheochromocytoma-induced cardiogenic shock	9
G. Rojas-Marte, J. John, A. Sadiq et al.	Cardiovasc Revasc Med	2015	Medication-induced Takotsubo Cardiomyopathy presenting with cardiogenic shock-utility of extracorporeal membrane oxygenation (ECMO): case report and review of the literature	1
H. Sumida, K. Morihisa, K. Katahira et al.	Intern Med	2017	Isolated Right Ventricular Stress (Takotsubo) Cardiomyopathy	1
H. Zhang and X. Liao	J Card Surg	2021	Takotsubo cardiomyopathy following pericardiectomy: A case report	1
I. Schroeder, M. Zoller, M. Angstwurm et al.	nt J Artif Organs	2017	Venlafaxine intoxication with development of Takotsubo cardiomyopathy: successful use of extracorporeal life support, intravenous lipid emulsion and CytoSorb^®^	1
J. Feghaly, Z. Oman, D. Das et al.	Cureus	2021	Recurrent Stress-Induced Cardiomyopathy With Cardiogenic Shock Requiring Impella Left Ventricular Assist Device	1
J. H. Choi, I. D. Oh, E. Shin et al.	Acute Crit Care	2020	Extracorporeal membrane oxygenation for Takotsubo cardiomyopathy that developed after mitral valve replacement	1
J. J. J. Aalberts, T. J. Klinkenberg, M. A. Mariani et al.	Eur Heart J	2017	Mechanical circulatory support for refractory cardiogenic shock in Takotsubo syndrome: a case report and review of the literature	1
J. Kirigaya, N. Iwahashi, R. Tanaka et al.	Cureus	2022	A Fatal Case of Takotsubo Cardiomyopathy Secondary to Refractory Hypoglycemia in Severe Starvation: An Autopsy Case Report	1
J. Mierke, T. Loehn, A. Linke et al.	Eur Heart J Case Rep	2019	Reverse Takotsubo cardiomyopathy- life-threatening symptom of an incidental pheochromocytoma: a case report	1
J. O’Brien, S. Mahony, R. J. Byrne et al.	Eur Heart J Case Rep	2021	Dynamic left ventricular outflow tract gradient resulting from Takotsubo cardiomyopathy ameliorated by intra-aortic balloon pump counterpulsation: a case report	1
J. Wei, L. Zhang, X. Ruan et al.	Front Cardiovasc Med	2022	Case Report: Takotsubo Syndrome Induced by Severe Anaphylactic Reaction During Anesthesia Induction and Subsequent High-Dose Epinephrine Resuscitation	1
K. T. Webster, T. Apridonidze, P. R. Mopala et al.	Can J Cardiol 2019	2019	Stress-Induced Cardiomyopathy Complicated by Dynamic Left Ventricular Outflow Obstruction, Cardiogenic Shock, and Ventricular Septal Rupture	1
K. X. Fu, B. H. Z. Ng and M. H. X. Chua	BMC Pediatr	2019	A unique case of acute brain haemorrhage with left ventricular systolic failure requiring ECMO	1
K. Yamane, H. Hirose, G. R. Reeves et al.	J Heart Valve Dis	2011	Left ventricular dysfunction mimicking Takotsubo cardiomyopathy following cardiac surgery	1
L. C. Napp, R. Westenfeld, J. E. Møller et al.	Cardiovasc Revasc Med	2022	Impella Mechanical Circulatory Support for Takotsubo Syndrome With Shock: A Retrospective Multicenter Analysis	16
L. Paton and I. Quasim	Br J Anaesth	2013	Takotsubo cardiomyopathy: issues for the intensivist	1
L. Wert, J. Kempfert, V. Falk et al.	Interdiscip Cardiovasc Thorac Surg	2023	Transaxillary implantation of a temporary microaxial left ventricular assist device in a patient with a rectangular kinked subclavian artery	1
M. Bonacchi, A. Vannini, G. Harmelin et al.	Interact Cardiovasc Thorac Surg	2015	Inverted-Takotsubo cardiomyopathy: severe refractory heart failure in poly-trauma patients saved by emergency extracorporeal life support	4
M. Bonacchi, S. Valente, G. Harmelin et al.	Artif Organs	2009	extracorporeal life support as ultimate strategy for refractory severe cardiogenic shock induced by Tako-tsubo cardiomyopathy: a new effective therapeutic option	1
M. Hanif, M. A. Haider, Q. Xi et al.	Cureus	2020	Takotsubo Cardiomyopathy Triggered by the Death of Pets (Cats): Two Case Reports	1
M. Husaini, J. N. Baker, S. Cresci et al.	JACC Case Rep	2022	Recurrent Takotsubo Cardiomyopathy in a Patient With Hypertrophic Cardiomyopathy Leading to Cardiogenic Shock Requiring VA-ECMO	1
M. Moguilevitch, M. Rufino, J. Leff et al.	Liver Transpl	2015	Novel approach for heart failure treatment after liver transplantation	1
M. Nakamura, M. Nakagaito, M. Hori et al.	J Artif Organs	2019	A case of Takotsubo cardiomyopathy with cardiogenic shock after influenza infection successfully recovered by IMPELLA support	1
M. P. Silva, E. M. Vilela, R. L. Lopes et al.	Rev Port Cardiol	2015	Cardiogenic shock induced by Takotsubo cardiomyopathy: A new therapeutic option	1
O. Kiamanesh, E. N. Vu, D. L. Webber et al.	JACC Case Rep	2019	Pheochromocytoma-Induced Takotsubo Syndrome Treated With Extracorporeal Membrane Oxygenation: Beware of the Apical Sparing Pattern	1
P. A. Cotinet, P. Bizouarn, F. Roux et al.	Heart Lung	2021	Management of cardiogenic shock by circulatory support during reverse Tako-Tsubo following amphetamine exposure: A report of two cases	2
R. Dalla Pozza, A. Lehner, S. Ulrich, M. Nabauer et al.	World J Pediatr Congenit Heart Surg	2020	Takotsubo Cardiomyopathy Complicating Percutaneous Pulmonary Valve Implantation in a Child	1
R. Gurreri, P. Poommipanit and A. Alghamdi	Oxf Med Case Reports	2023	Cardiogenic shock secondary to stress-induced cardiomyopathy precipitated by severe diabetic ketoacidosis	1
R. Hamdan, M. E. Nassef, J. Khan et al.	Ann Cardiol Angeiol	2022	Reverse Takotsubo ou myocardite fulminante ? Succes de VA ECMO chez une patiente ayant une atteinte cardiaque liee COVID 19	1
R. Kakizaki, N. Bunya, S. Uemura et al.	Acute Med Surg	2019	Takotsubo cardiomyopathy developed during rewarming of accidental hypothermia with extracorporeal membrane oxygenation	1
R. Korabathina, W. Abel and A. Labovitz	Case Rep Cardiol	2016	Cardiogenic Shock due to Psychosis-Induced Inverted Takotsubo Cardiomyopathy Bridged-to-Recovery with a Percutaneous Left Ventricular Assist Device	1
R. Nishikawa, N. Nagano, N. Kokubu et al.	Int Heart J	2021	Favorable Effects of Impella on Takotsubo Syndrome Complicated with Cardiogenic Shock	4
R. S. Biondi, V. S. Barzilai, A. L. C. Watanabe et al.	Rev Bras Ter Intensiva	2018	Use of extracorporeal membrane oxygenation for treating acute cardiomyopathy after liver transplantation: a case report	1
R. V. Reddy, S. Agarwal, V. Choudhary et al.	Indian J Anaesth	2018	Reverse stress cardiomyopathy post-liver transplant needing mechanical circulatory support	1
S. An, H. I. Ma, J. Song et al.	*BMC Neurol*	2020	Stress cardiomyopathy associated with area postrema syndrome as a presentation of neuromyelitis optica: case report	1
S. Elapavaluru, A. Gologorsky, N. Thai et al.	J Cardiothorac Vasc Anesth	2017	Perioperative Stress Cardiomyopathy in Simultaneous Liver and Kidney Transplantation: A Call for Early Consideration of Mechanical Circulatory Support	1
S. Fang, Y. Wang, P. K. He et al.	Medicine (Baltimore)	2021	Cardiogenic shock caused by Takotsubo syndrome complicated with severe anxiety: A case report and literature review	1
S. Forsberg, L. Abazi and P. Forsman	J Med Case Rep	2021	Successful use of extended cardiopulmonary resuscitation followed by extracorporeal oxygenation after venlafaxine-induced Takotsubo cardiomyopathy and cardiac arrest: a case report	1
S. H. Park, M. K. Song, G. B. Kim et al.	Chonnam Med J	2019	Stress Induced Cardiomyopathy Requiring Ventricular Assist Device Support in an 8-Year-Old Girl with Acute Leukemia	1
S. Kaese, C. Schulke, D. Fischer et al.	Intensive Care Med	2013	Pheochromocytoma-induced Takotsubo-like cardiomyopathy and global heart failure with need for extracorporal life support	1
S. Kurisu, K. Ishibashi, Y. Kato et al.	Intern Med	2012	Tako-tsubo cardiomyopathy complicated by QRS prolongation	1
S. Lee, S. P. Lim, J. H. Yu et al.	Korean J Thorac Cardiovasc Surg	2011	Stress-induced Cardiomyopathy during Pulmonary Resection (Takotsubo Syndrome)—A case report	1
S. Li, M. M. Koerner, A. El-Banayosy et al.	Ann Thorac Surg	2014	Takotsubo’s syndrome after mitral valve repair and rescue with extracorporeal membrane oxygenation	1
S. Modi and D. Ramsdale	Int J Cardiol	2011	Tako-tsubo, hypertrophic obstructive cardiomyopathy & muscle bridging--separate disease entities or a single condition?	1
S. Park, M. Kim, D. I. Lee et al.	Acute Crit Care	2022	Successful extracorporeal membrane oxygenation treatment of catecholamine-induced cardiomyopathy-associated pheochromocytoma	1
S. Sossalla, C. Meindl, M. Fischer et al.	Circ Cardiovasc Interv	2019	Bail-Out Alcohol Septal Ablation for Hypertrophic Obstructive Cardiomyopathy in a Patient With Takotsubo Cardiomyopathy-Induced Cardiogenic Shock	1
S. Sundaravel, A. Alrifai, M. Kabach et al.	Case Rep Cardiol	2017	FOLFOX Induced Takotsubo Cardiomyopathy Treated with Impella Assist Device	1
T. Attisano, A. Silverio, C. Prota et al.	ESC Heart Failure	2020	Impella in Takotsubo syndrome complicated by left ventricular outflow tract obstruction and severe mitral regurgitation	1
T. Bleser, C. Weth and G. Gorge	Med Klin Intensivmed Notfmed	2013	Reverse Takotsubo cardiomyopathy-a life-threatening disease. Successful resuscitation of a 31-year-old woman with cardiologic shock after a visit to the dentist	1
T. E. Pearson, M. A. Frizzola, M. A. Priest et al.	Air Med J	2018	Pediatric Extracorporeal Cardiopulmonary Resuscitation Patient With Traumatic Subarachnoid Hemorrhage and Takotsubo Syndrome	1
T. K. Yoo, J. Y. Lee, K. C. Sung et al.	J Cardiovasc Ultrasound	2016	Stress-Induced Cardiomyopathy Presenting as Shock	1
T. Lyu, J. Niu, Z. Liu et al.	Front Cardiovasc Med	2022	Case Report: Early Resection of Pheochromocytoma in a Patient With Cardiogenic Shock Due to Pheochromocytoma-Induced Cardiomyopathy With Extracorporeal Life Support	1
V. V. Garla, S. Gosi, S. Kanduri et al.	BMJ Case Rep	2019	A case of catecholamine-induced cardiomyopathy treated with extracorporeal membrane oxygenation	1
X. Fan, P. Liu and L. Bai	Eur Heart J Case Rep	2022	Cardiogenic shock due to Takotsubo cardiomyopathy associated with thyroid crisis: a case report	1
Y. Luo, X. Ye, L. Zhang et al.	Echocardiography	2023	A rare case of cardiogenic shock caused by Takotsubo syndrome associated with SARS-CoV-2 infection: The value of echocardiography in the diagnosis and monitoring of the efficacy of extracorporeal membrane oxygenation	1
Y. Xie, A. Zhang, M. Qi et al.	BMC Endocr Disord	2023	Pheochromocytoma crisis with refractory Acute Respiratory Distress Syndrome (ARDS), Takotsubo syndrome, emergency adrenalectomy, and need for Extracorporeal Membrane Oxygenation (ECMO) in a previously undiagnosed and asymptomatic patient, due to the use of metoclopramide	1
Y. Y. Jo, S. Park and Y. S. Choi	Anaesth Intensive Care	2011	Extracorporeal membrane oxygenation in a patient with stress-induced cardiomyopathy after caesarean section	1
Z. Su, Y. Wang and H. Fei	CASE (Phila)	2019	Takotsubo-Like Cardiomyopathy in Pheochromocytoma	1
Z. Y. Zhang, J. J. Sun, J. H. Wang et al.	BMC Cardiovasc Disord	2023	Successful treatment of a severe Takotsubo syndrome case complicated by liver abscess	1
